# Microstructure and Its Influence on the Welding Quality of 6063 Aluminum Alloy Porthole Die Extrusion

**DOI:** 10.3390/ma14216584

**Published:** 2021-11-02

**Authors:** Shikang Li, Luoxing Li, Zhiwen Liu, Guan Wang

**Affiliations:** 1 School of Science and Engineering, Huzhou College, Huzhou 313000, China; 2 State Key Laboratory of Advanced Design and Manufacturing for Vehicle Body, Hunan University, Changsha 410082, China; luoxingli@hnu.edu.cn; 3 College of Mechanical Engineering, University of South China, Hengyang 421001, China; liuzhiwen1008@163.com; 4 School of Mechanical Engineering, Ningxia Universitya, Yinchuan 750021, China; belonging1024@126.com

**Keywords:** ram speed, dynamic recrystallization, weld quality, porthole die extrusion

## Abstract

Extrusion experiments and 3D numerical modeling were conducted to investigate the dynamic recrystallization and welding quality of a 6063 aluminum alloy hollow square tube extruded by a porthole die at the ram speeds of 3 mm/s, 7 mm/s, 9 mm/s and 11 mm/s. The results showed that average grain size of hollow square tube extruded at the ram speed of 7 mm/s was the smallest. The profile extruded at the ram speed of 3 mm/s exhibited the highest expansion ratio. Dynamic recrystallization (DRX) fractions were highly variable at different ram speeds. DRX fractions in the matrix zones were higher than those in the welding zones, resulting in smaller grain sizes in the matrix zones. Mechanical properties in the welding zones and matrix zones was different. A local strain concentration would occurred during expansion, which would affect the welding quality. Finally, it was found that the uniform microstructure near the welding line would also affect the welding quality.

## 1. Introduction

The usage of lightweight and high strength materials, such as Al alloy hollow profiles, was growing. Most Al alloy hollow profiles were fabricated by porthole die extrusions. During the process of porthole die extrusion, one or more longitudinal weld seams are formed [1]. The weld seams or near zones are the weakest zones of the profile [2], where a fracture is prone to occur when the profile is in a corrosive environment or subjected to external stress. Therefore, investigation into the welding quality of the longitudinal seam is essential.

Researchers have proposed several criteria to evaluate welding quality [3,4,5]. Akeret [3] found that the maximum pressure on the welding surface must exceed a critical value to ensure welding quality and proposed the P criterion. Plata and Piwnik [4] put forward the Q criterion according to the effect of contact time on the welding strength and proposed that the Q value should exceed a critical limit to achieve a good welding quality. Depending on a large number of experiments and research, Donati and Tomesani [5] put forward the K criterion and found that a threshold value must be reached to obtain good welding quality. Although these criteria have been effectively applied to evaluate welding quality, the welding process is complex during porthole die extrusion. The welding quality depends not only on the thermodynamic parameters of the welding surface but also on microstructures near the welding seam.

Occurrence of DRX would influence the microstructures and mechanical properties of welding seam. Chen et al. [6] found that the volume fraction of DRX was difference in different zones during porthole die extrusion. The inhomogeneous DRX behavior led to the variable distribution of strain on the extrusion profile, which was unfavorable to welding quality.

The Al/Mg/Al laminate was fabricated through a porthole die co-extrusion [7]. Partial DRX and an approximately complete DRX occurred in the Al layer and Mg layer, respectively. In addition, the hardness of the Al/Mg interface was lower than that of the matrix zone due to a higher DRX degree. Fan et al. [8] studied the development of grains and their orientations and found that DRX was one of the grain refinement mechanisms. Yu et al. [9,10,11] found that DRX in the welding zone was advantageous to the evolution of low angle grain boundaries and grain refinement. The decrease of grain size contributed to the improvement of mechanical properties.

In summary, although much research has investigated welding quality, there are still problems needing further investigation and clarification, such as the effect of the microstructure on welding quality. Zhang et al. [12] found that the microstructure had an influence on the weld quality based on the results of tensile strength and J values. However, there was no further explanation. In this work, 3D numerical modeling was utilized to evaluate the welding quality using the traditional K criterion. Moreover, the porthole die extrusions were carried out for a 6063 aluminum hollow tube. The microstructures of the profiles were characterized and analyzed. The grain sizes and DRX fractions in the welding zones and matrix zones were calculated. The mechanical properties of the 6063 aluminum alloy porthole die extrusions were tested. Based on the above work, the welding quality of a 6063 aluminum alloy hollow tube was investigated.

## 2. Experimental Procedures

### 2.1. Materials and Extrusion

**Figure 1:**
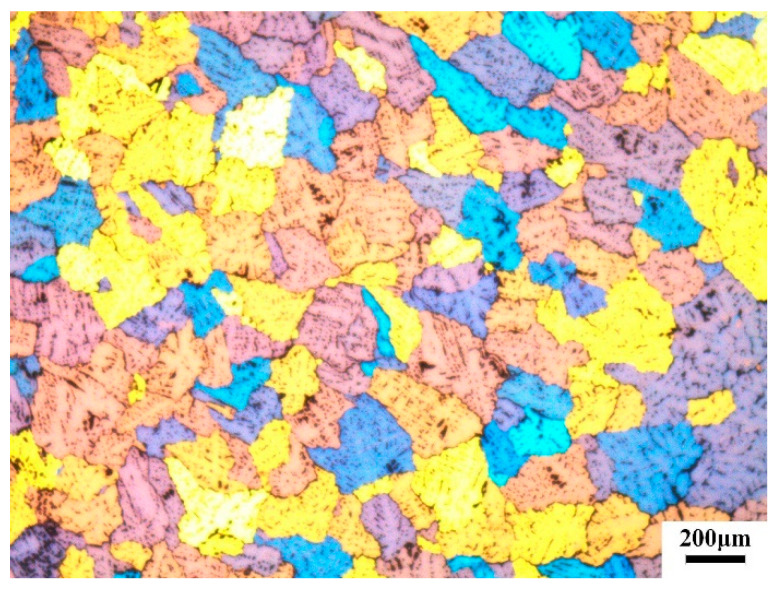
Base material microstructure before extrusion.

The chemical composition (wt%) of the studied 6063 aluminum alloy was Al-0.85Mg-0.47Si-0.2Fe-0.21Cu-0.01Mn-0.13Cr-0.25-Zn-0.15%Ti. The 6063 aluminum alloy billets were homogenized at 540 °C for 24 h and then air-cooled to room temperature. Uniform grain structures were observed in the homogenized materials and the average grain size was around 198 μm according to ASTM E1382, as shown in Figure 1.

**Figure 2:**
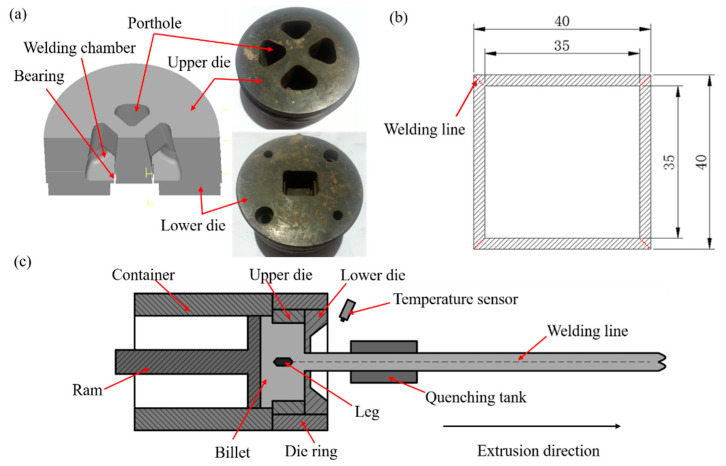
(**a**) Porthole die, (**b**) cross-section shape of the profile, and (**c**) schematic of the extrusion process (unit: mm).

Before extrusion, the homogenized 6063 aluminum billets were cut and machined to Ø86 × 300 mm. The extrusion experiment was carried out on an 800 ton extruder at ram speeds of 3 mm/s, 7 mm/s, 9 mm/s, and 11 mm/s, respectively. The extrudate temperature was measured and recorded by a 3T temperature sensor. The SD3000 data acquisition and analysis software application was used in conjunction with the 3T pyrometer system. The extrusion ratio of porthole die was 16.9, as shown in Figure 2a. After extrusion, the profiles were quenched into water for preservation of extrudate microstructures. The profile was a square tube with an out length of 40 mm and a wall thickness of 2.5 mm, as shown in Figure 2b. The welding seams were located in the corners of the square hollow tube. The setup of designed experiment and the material flow behavior were shown in Figure 2c.

### 2.2. Mechanical FTests

A Vickers hardness tester was loaded with 0.5 kgf and the dwell time was 15 s during the measurement of hardness. The distance between successive indentations was 100 μm. In addition, the hardness testing was performed on the cross direction of the profile. The cross direction surface of extrusion profile was used for hardness test. The HV0.5 values were averages of at least 7 indentations.

**Figure 3:**
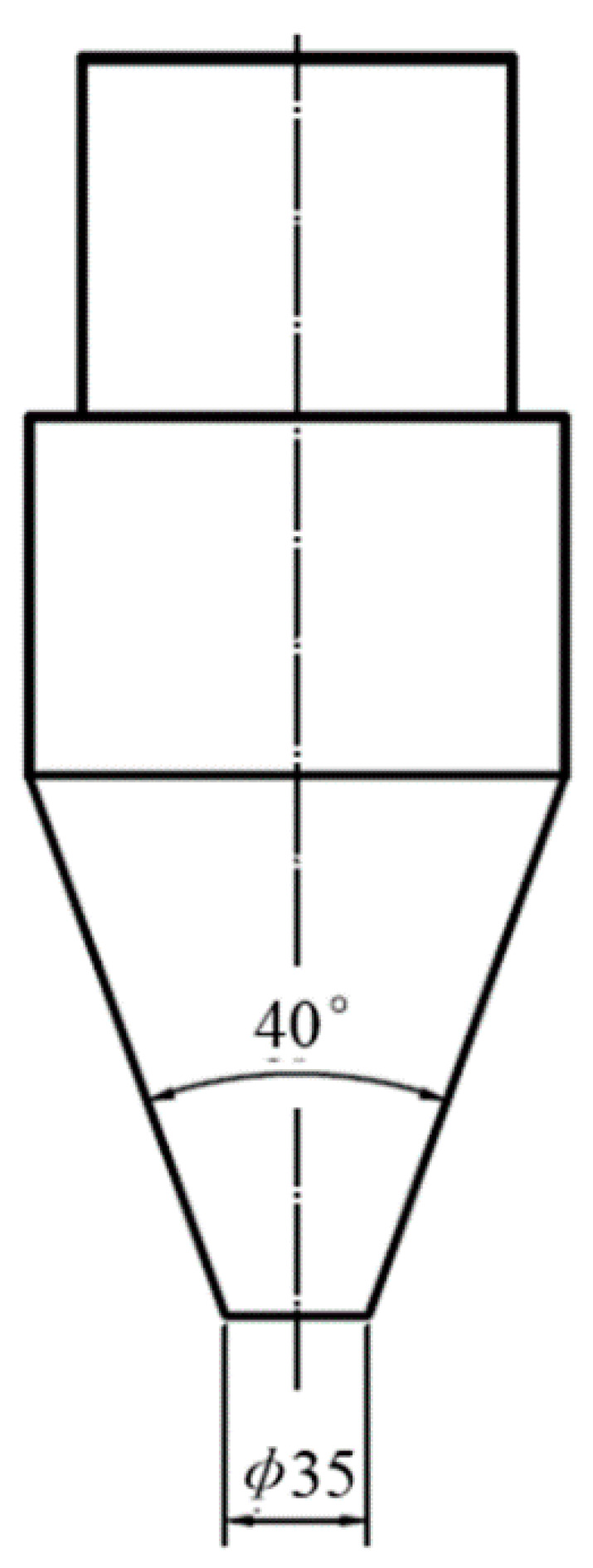
Conical mandrel dimensions in the expansion test (unit: mm).

To determine the longitudinal seam quality, a conical punch was employed during expansion test. The experimental setup was designed according to the methodology proposed by Li et al. [13]. A conical punch which was forced to move inside the 6063 aluminum alloy profile until the crack appeared, as shown in Figure 3. The expansion test was carried out at a compression rate of 2 mm/min by an Instron 3369 electromechanical machine.

### 2.3. Microstructural Characterization

The microstructures were observed by optical observation (OM). For the OM observation, the observed surfaces of the samples were first electropolished in a solution of 10 mL HCLO_4_ and 90 mL C_2_H_6_O at 25 V for 8 s. Then, anodic coatings were performed on the same surfaces. A mixed solution consisting of 5 g HBF4 and 200 mL water was used. The time of anodic coatings was 3 min.

The DRX behaviors of the extrudate profiles were observed by a ZEISS EVO MA10 scanning electron microscope (SEM) equipped with an electron backscattered diffraction (EBSD) detector. The samples were electropolished with 7 vol. % HClO_4_ acids in alcohol at a voltage of 25 V for 6 s. The step size of EBSD observation was 2 μm. The post-EBSD data processing was performed on the commercial software HKL Channel 5.

### 2.4. Finite Element Method

The numerical model was established with DEFORM-3D software. In the simulation, the billet and tooling were set as a thermo-viscoplastic model and a thermo-rigid model, respectively. Hot compressions were performed using a Gleeble-1500 thermo-mechanical simulator to obtain stress–strain curves over a temperature ranging from 573 to 773 K and a strain rate ranging from 0.01 to 10 s^−1^ with a true strain of 0.9. The friction factor at the interfaces between the billet and tools was set as 0.4 according to the system setting of DEFORM software. Due to the symmetry of porthole die and reducing the computational time, one-eighth of the numerical simulation model was set. The porthole areas of the die and container were meshed into tetrahedral elements with a size of 5 mm and 1mm, respectively. Others were meshed into tetrahedral elements with a size of 0.3 mm.

## 3. Results and Discussion

### 3.1. Theoretical Analysis of the Welding Quality

In this section, the welding quality of 6063 aluminum square tubes was studied based on the *K* criterion, which is in accordance with the integral in the welding path of the ratio of welding pressure to the effective stress of the billet, and can be expressed as [5]:

(1) $$K=\int_{t}\frac{p}{\sigma }dt\cdot v=\int_{L}\frac{p}{\sigma }dl\geq Const$$

where *p* is the welding pressure, *σ* is the effective stress of the billet, *L* is welding path from the end point of the die bridge up to the die exit, and *Const* is a threshold value to determine the welding strength. This value is a constant under the given porthole die and material [14].

**Figure 4:**
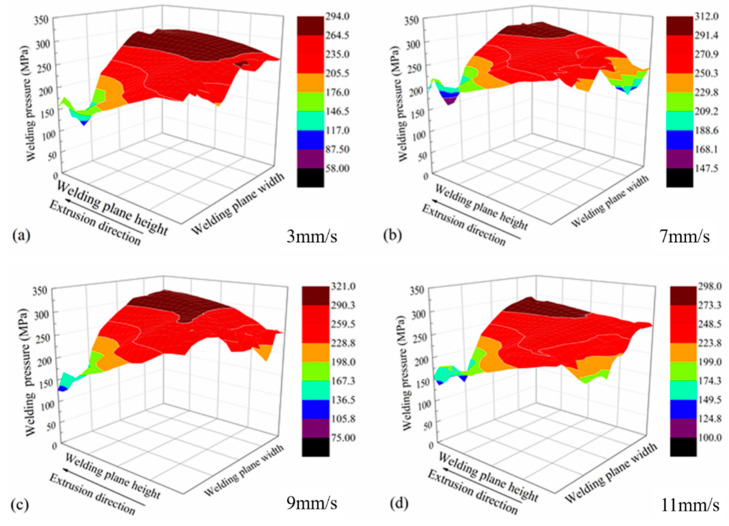
Welding pressure distributions on the welding planes. (**a**–**d**) different ram speed.

Figure 4 shows the distributions of the pressures on the welding planes. The welding pressures increased while the ram speed increased from 3 mm/s to 9 mm/s. However, while the ram speed increased to 11 mm/s, the welding pressure slightly decreased. This was due to a higher temperature rise at a higher ram speed. In addition, the welding pressure inside the welding chamber was lower than that in the outside part.

**Figure 5:**
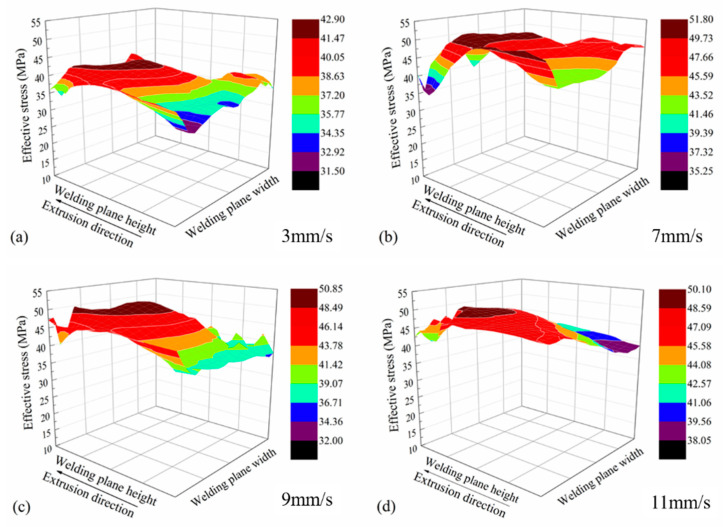
Effective stress distributions on the welding planes. (**a**–**d**) different ram speed.

Figure 5 shows the distributions of effective stresses on the welding planes. The effective stress increased with the increase in ram speed. The maximum effective stress on the welding plane was 42 MPa at the ram speed of 3 mm/s and 50 MPa at the ram speed of 11 mm/s, respectively.

**Figure 6:**
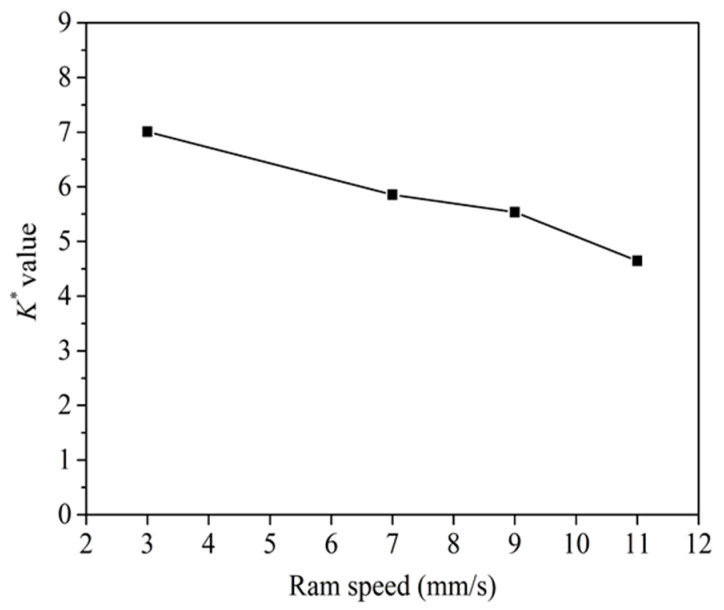
The relationship between the ram speed and K* value.

According to Equation (1), the *K* values at different ram speeds were calculated. According to the study of Lu et al. [15], three-dimensional values of *K* can be transformed into nondimensional ones (K*) by dividing the area of the welding plane. Figure 6 shows the nondimensional K* values. The K* values decreased with the increase in ram speed, which was in accordance with the experimental results of literatures [16,17]. Donati and Tomesani [5] suggested that *K* value on the welding surface should exceed 3 or 4 to ensure the welding quality. Based on the above calculation, the K* values all exceeded 4, which indicated that the solid-state welding had taken place.

### 3.2. Microstructure of the Extrudate Profiles

**Figure 7:**
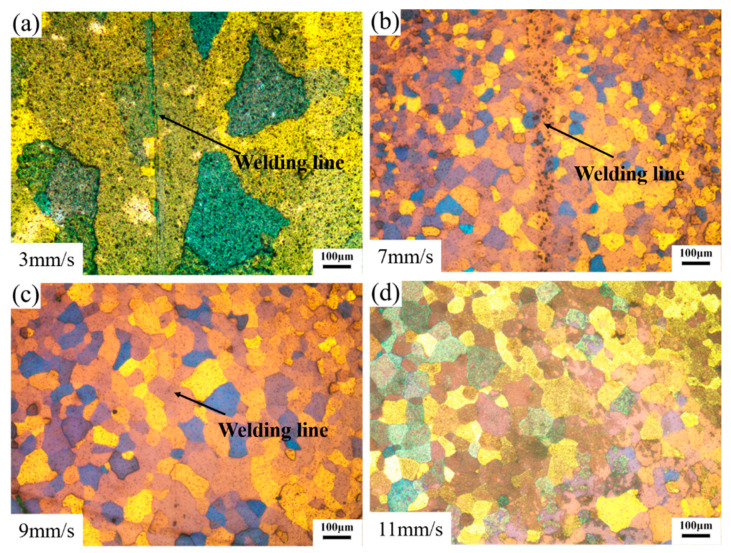
Microstructures of the welding zones on the transverse cross-section. (**a**–**d**) different ram speed.

Figure 7 shows the microstructures of the welding zones on the transverse cross-section of the extrudate profiles. The grains in the welding zone of the profile extruded at the ram speed of 3 mm/s had the largest size, as shown in Figure 7a. The grain sizes in the welding zones of profile extruded were refined, as shown in Figure 7b–d. Fine equiaxed grains were observed around the welding lines. Moreover, the grain size increased slightly with the increase in ram speed from 7 mm/s to 11 mm/s. Finally, according to the OM maps, there were no micro-voids around the weld line and a sound weld seam was achieved.

**Figure 8:**
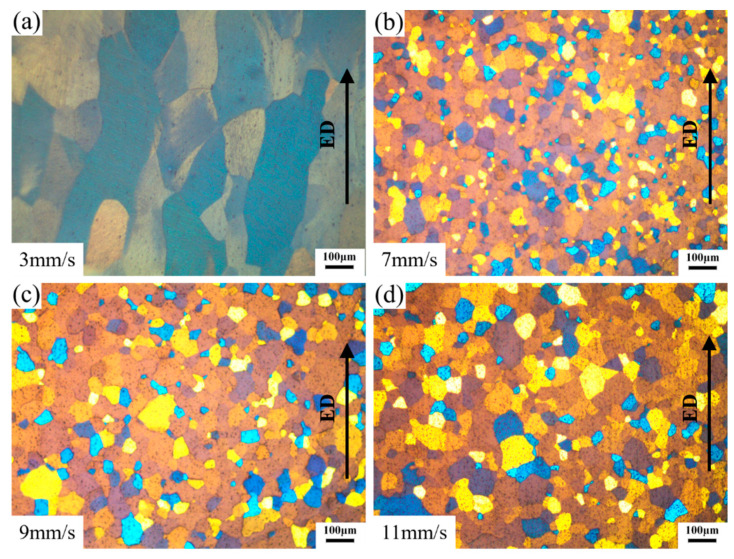
Microstructures of the extruded material on longitudinal cross-section. (**a**–**d**) different ram speed.

Figure 8 shows the microstructures of extruded profiles on the longitudinal cross-section at different ram speeds. A few bands of elongated grains along the extrusion direction (ED) were observed on the profile extruded at a ram speed of 3 mm/s, as shown in Figure 8a. Equiaxed grain structures were obtained for the profiles extruded at the ram speeds of 7 mm/s, 9 mm/s, and 11 mm/s, as shown in Figure 8b–d. It indicated that DRX occurred during porthole die extrusion. Moreover, some fine grains were observed around the coarse grains at the profiles extruded at the ram speeds of 7 mm/s, 9 mm/s, and 11 mm/s.

**Figure 9:**
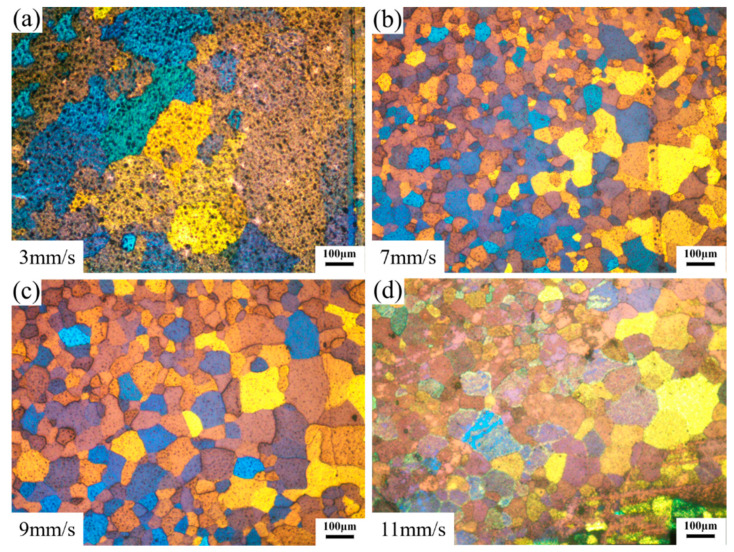
Microstructures of the matrix zones on the transverse cross-section. (**a**–**d**) different ram speed.

Figure 9 shows the microstructures of the matrix zones on the transverse cross-section of profiles extruded at different ram speeds. Obviously, the grain sizes in the matrix zones were fine compared with those in the welding zones. At the ram speed of 3 mm/s, the grains in the matrix zone had the largest size and some fine grains were also observed, as shown in Figure 9. Compared with the grains in the matrix zone of the profile extruded at a ram speed of 3 mm/s, the grains of the profile extruded at a ram speed of 7 mm/s, 9 mm/s, and 11 mm/s were much finer, as seen in Figure 9b–d. Moreover, the grain sizes slightly increased with the increase in ram speed from 7 mm/s to 11 mm/s.

**Figure 10:**
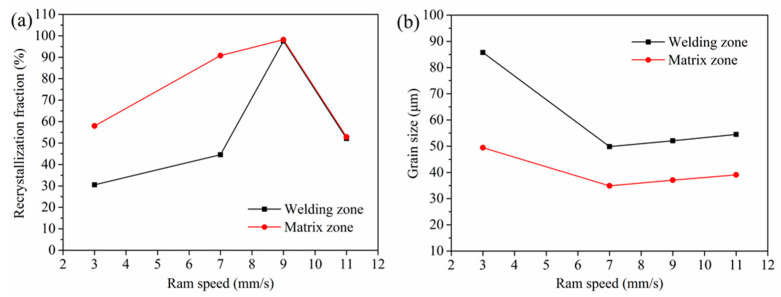
(**a**) DRX fractions and (**b**) grain sizes of porthole die extrusions at different ram speeds.

Figure 10a displays the DRX fraction in the matrix zones and welding zones at different ram speeds. The DRX fraction in the matrix zone of the profile increased nearly linearly with the increase in ram speed from 3 mm/s to 9 mm/s. It may be that the dynamic recovery (DRV) preceded the DRX at a low strain rate for aluminum alloy [18]. Further, to increase the strain rate, the absence of DRV provided enough stored energy for DRX. Due to insufficient time for the growth of the misorientation among the subgrains, the DRX fraction decreased at the ram speed of 11 mm/s. The evolution of subgrains to viable recrystallization nuclei was suppressed.

Compared with the matrix zone, the DRX behavior in the welding zone was restricted. At the ram speed of 3 mm/s, a relatively low DRX fraction was observed. An approximately complete DRX occurred in the profile extruded at the ram speed of 9 mm/s. At the ram speeds of 3 mm/s, 7 mm/s, 9 mm/s, and 11 mm/s, the DRX fractions in the welding zone were 30.5%, 44.5%, 97.5%, and 52.1%, respectively. While the DRX fraction was 57.9%, 90.7%, 98.1%, and 52.8%, away from the welding zone, respectively. The different DRX fractions in the different zones of the 6063 aluminum alloy profile may be due to different strain rates. There was sufficient time for growth of the misorientation among the subgrains away from the welding zone due to the low strain rate. That was advantageous to the evolution of subgrains to viable recrystallization nuclei.

The average grain sizes of the extrudate profiles were measured according to the ASTM-E1382 linear intercept length method in two evenly spaced directions (0° and 90°). Figure 10b displays the grain size in the matrix zones and welding zones at different ram speeds. Increasing ram speed caused a sharp decrease in grain size at an initially low speed due to DRX. In contrast, at a high ram speed, the grain size increased due to the increased extrudate temperature. It was found that grain size increased nearly linearly with the increase in ram speed from 7 mm/s to 11 mm/s. The grain size in the matrix zone was lower than that away from the welding zone due to a large DRX fraction. At the ram speeds of 3 mm/s, 7 mm/s, 9 mm/s, and 11 mm/s, the grain size was 86 μm, 49 μm, 52 μm, and 54 μm in the welding zone, respectively. Away from the welding zone, the grain size was 49 μm, 34 μm, 37 μm and 39 μm, respectively. Compared with the grain size of the 6063 aluminum alloy homogenized billet, the grain size of the extrudate profile was obviously refined, which was the result of DRX.

### 3.3. Evolution of the Welding Quality

**Figure 11:**
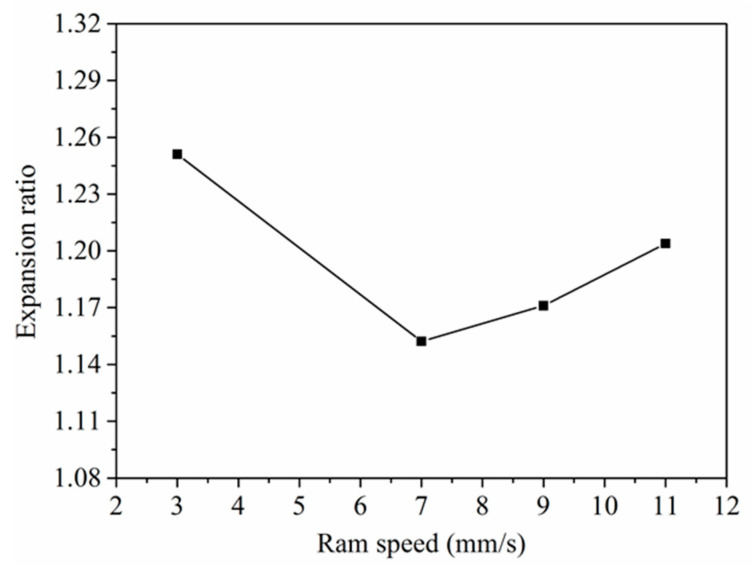
The relationship between the ram speed and expansion ratios.

According to the results of the *K* criterion, the welding quality of the 6063 aluminum tubes could be ensured during porthole die extrusion. Figure 11 shows the expansion ratios of the 6063 aluminum square tubes at different ram speeds. The expansion ratio firstly decreased and then showed an increase with the increase in ram speed. At the ram speeds of 3 mm/s, 7 mm/s, 9 mm/s, and 11 mm/s, the expansion ratio obtained for the 6063 aluminum alloy hollow square tube was 1.25, 1.15, 1.17, and 1.20, respectively. The expansion results showed that ram speed had a significant influence on the welding quality of the 6063 aluminum alloy square tube.

As stated earlier, the welding seam formation is a solid-state bonding process [15]. Diffusion mechanisms contribute to the bond strength. At a low ram speed, there was much time for the movement of the atoms in the aluminum alloys, resulting in high welding strength. There was no enough time for diffusion to proceed a high ram speed, which was adverse to welding behavior [19]. Thus, the expansion ratio decreased from 1.25 to 1.15 with the ram speed from 3 mm/s to 7 mm/s. However, the expansion ratio showed an increasing trend, with the increase in ram speed from 7 mm/s to 11 mm/s. This might be due to the uniformity of the extrudate microstructure.

In the porthole die extrusion, the microstructure between the welding zone and matrix zone was different [6,7,8,9]. According to the study of He et al. [20], if the microstructure was composed of two different grain sizes in two adjacent zones, deformation often propagated quickly and the material easily failed catastrophically at the interface because of the stress concentration. During the 6063 aluminum porthole die extrusions, the higher DRX in the matrix zone led to the smaller grain size.

**Figure 12:**
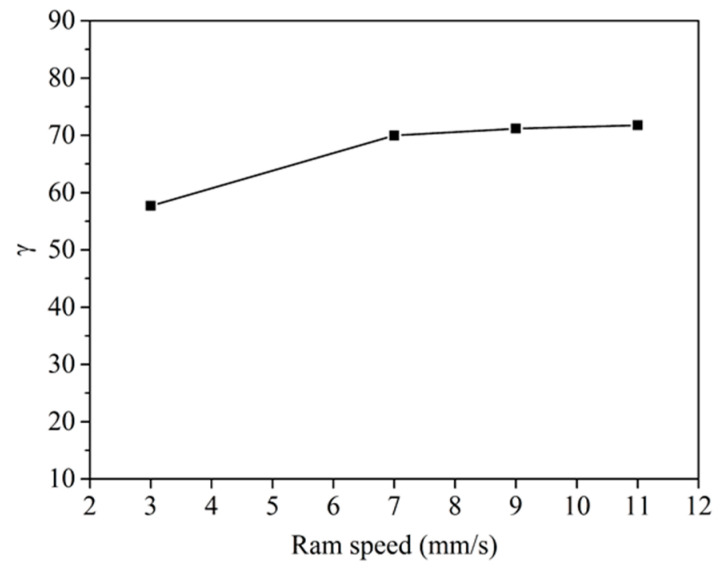
The relationship between the ram speed and *γ* value.

To determine the uniformity of the microstructure of the profile, *γ* was defined as:

(2) $$\gamma =\frac{d_{m}}{d_{w}}\times 100$$

where *d*_m_ and *d_w_* are the average grain sizes in the matrix zone and welding zone, respectively. The closer the value of *γ* to 100, the more uniform the microstructure is. Figure 12 shows the values of *γ*. The value of *γ* increased with the increase in ram speed from 3 mm/s to 7 mm/s. It indicated that increasing speed was beneficial to improve the microstructure uniformity in the porthole die extrusion profile.

The different grain sizes in the welding zone and matrix zone would cause different mechanical properties. According to the work of Hall and Petch [21], the mechanical properties of metallic materials have been shown to correlate with the grain size. A relationship was found between the yield strength of metallic materials and grain size. Up to now, the Hall–Petch relationship has been applied to a large variety of metallic materials and their properties, for example, fatigue and microhardness. In general, the yield strength could be replaced by microhardness (*H_v_*) and the Hall–Petch relationship can be described as:

(3) $$H_{v}=H_{vo}+k\cdot d^{-1/2}$$

where *H_vo_* is the hardness of a single crystal and *k* is the Hal–Petch slope of the microhardness.

**Figure 13:**
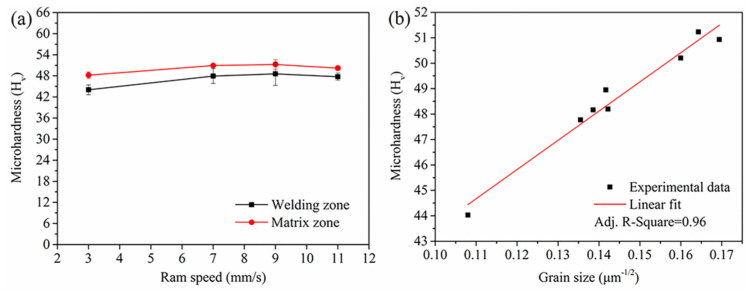
Microhardness of profile as a function of (**a**) ram speed, (**b**) average grain size.

Figure 13a shows the microhardness values of the porthole die extrusion profiles. The average microhardness of the as-cast billet was found to be 38 ± 3 HV0.5. It was found that the extrusion process improved the microhardness of the extruded profiles. The microhardness of the extrudate profile firstly increased and then decreased. This was because the high extrudate temperature resulted in grain coarsening and caused the decreased microhardness at a high speed. At the ram speed of 3 mm/s and 9 mm/s, the microhardness of the profile had the lowest and largest values, respectively. In general, the microhardness value of the zone far away from the welding zone was larger than that in the welding zone.

Stathers et al. [22] found that microhardness could be used as a robust characterization tool to forecast as the yield strength (YS) and the ultimate tensile strength (UTS) through experiments Figure 13b shows the microhardness value as a function of the inverse square root of grain sizes. The microhardness values exhibited a very reasonable linear relationship with the inverse square root of the grain sizes as evidenced by the Adj. R-Square value of 0.96. The best fitting relationship indicated that the grain size dependence of microhardness complied with the Hall–Petch relationship.

The smaller grain size in the zone far away form the welding zone resulted in higher yield stress and microhardness. The strength contributed by the grain refinement can be described by the Hall–Petch relationship [23]:

(4) $$\Delta \sigma_{H–P}=\frac{K_{y}}{\sqrt{D}}$$

where Δ*σ_H–P_* is the increment of yield strength contributed from grain refinement, *K_y_* is constant of order 0.25 MPa $\sqrt{m}$ for the Al alloy, and *D* is the average grain size for the alloy.

**Figure 14:**
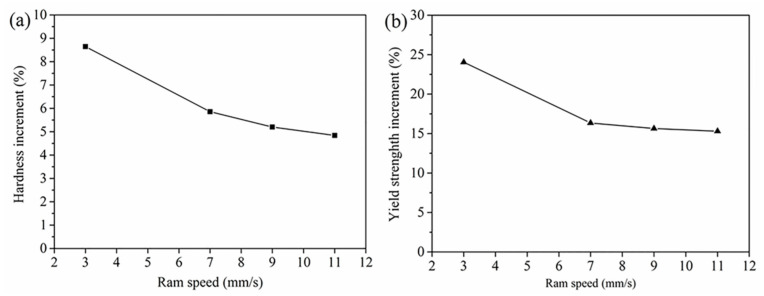
Increments of mechanical properties due to smaller grain sizes in the matrix zones: (**a**) microhardness increment; (**b**) yield strength increment.

Figure 14 shows the percentages of the increments of microhardness and yield strength in the matrix zones. Overall, the increment of microhardness and yield strength showed a declining trend with the increase in ram speed. It meant that the gradient of microhardness and yield strength between the welding zones and matrix zones decreased with the increase in ram speed. That is, increasing extrusion speed can reduce the difference of mechanical properties in different zone of the extrusion profile.

The lower fraction of DRX led to grain coarsening in the welding zone and thus to a reduced microhardness and yield strength, as shown in Figure 13 and Figure 14. The softening character caused an intense strain localization at the transition zone between welding zone and matrix zone, or within the welding zone during expansion. A local strain concentration would lead to local crack initiation. The varying and complex local microstructure or yield strength in the weld zone affected both the crack initiation sites and the crack propagation paths [24,25].

Strong inhomogeneous microstructure was formed in the 6063 aluminum alloy hollow square tube during extrusion. As a result, the welding zone had the lowest hardness. Crack initiation and local yielding would take place at the transition zone between welding zone and matrix zone or within the welding zone during expansion. According to the study of Flipon et al. [26], the fracture was mostly intergranular and, as a consequence, triggered by the interfaces between coarse grains and fine grains. During expansion, the greater the difference of the mechanical properties between the welding zone and matrix zone was, the greater the local strain concentration was, leading to a decreasing expansion ratio.

Moreover, the microstructure uniformity is beneficial to alleviate strain localization and instability in plastic deformation [27]. At a high ram speed, the difference in the grain size, microhardness, and yield strength in 6063 aluminum alloy hollow square tube decreased (as shown in Figure 12 and Figure 14), resulting in the reducing local stress concentration or local strain concentration in the 6063 aluminum alloy hollow square tube during expansion. Therefore, the welding quality obtained for the 6063 aluminum alloy hollow square tube increased with the increase in ram speed from 7 mm/s to 11 mm/s.

**Figure 15:**
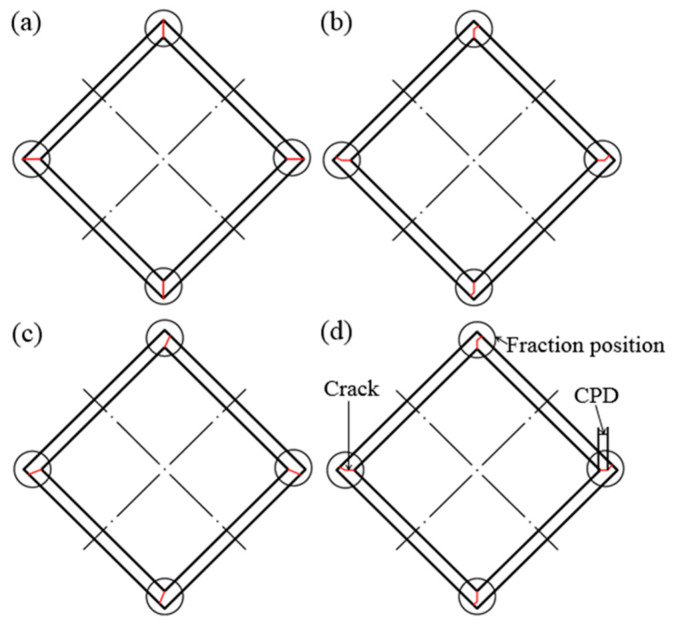
Schematic diagram of fracture model of expansion test: (**a**) Type I; (**b**) Type II; (**c**) Type III; (**d**) Crack propagation distance (CPD) along the weld line.

During the expansion test, there were three types of crack propagation for the square tube, i.e., Type I, Type II, and Type III crack propagations, shown in Figure 15. If a solid bonding was not achieved during the porthole die extrusion, the crack would propagate along the weld line during the expansion test, i.e., Type I, as shown in Figure 15a. In the case of a sound weld, the crack would tend to propagate to the geometric transition zone, i.e., Type II and Type III propagations, as shown in Figure 15b,c. If the microstructure of the porthole die extrusion was uniform, the crack propagation was likely to be Type III.

**Figure 16:**
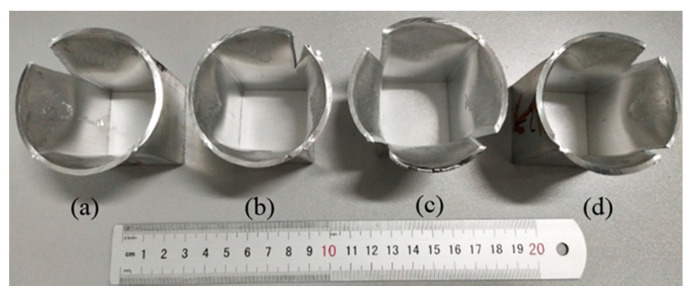
Fracture modes of expansion test samples at the ram speed of: (**a**) 3 mm/s; (**b**) 7 mm/s; (**c**) 9 mm/s; (**d**) 11 mm/s.

Figure 16 shows the fractured samples extruded at different ram speeds. Fractures occurred at the corners due to the weld seams and geometry of the square tube. In addition, the crack firstly propagated along the weld line and then turned to the geometric transition zone. Closer inspection of the fracture surface revealed that plastic deformation occurred during the expansion test. That is, a strong solid-state welding was achieved. The crack propagation distance along the weld line during the expansion test is shown in Table 1. It was found that the crack propagation along the weld line decreased from 0.81 mm to 0.66 mm with the increase in ram speed from 7 mm/s to 11 mm/s, which also indicated that the welding quality increased with the increase in ram speed from 7 mm/s to 11 mm/s.

According to the above discussion, different DRX fractions caused the different microstructures in the 6063 aluminum alloy hollow square tube. The gradient of grain sizes from the welding zone to the matrix zone caused local strain concentration or stress concentration at the interface of the welding zone and the matrix zone during expansion tests. The local strain concentration or stress concentration had a negative influence on the welding strength. The smaller the difference in the microstructure in the extrusion profile, the higher the welding quality. Namely, a uniform microstructure contributed to the welding quality.

## 4. Conclusions

In this study, the welding quality of a 6063 aluminum alloy hollow square tube extruded by a porthole die was studied. The *K* criterion was introduced to evaluate the welding quality. The DRX fraction, grain size, and its influence on the welding quality were analyzed. The main conclusions are drawn below:

1. According to the *K* criterion and microstructures in the welding zones, good solid-state welding was achieved in all conditions;
2. The fraction of DRX first increased with the increase in ram speed from 3 mm/s to 9 mm/s and then decreased with further increasing ram speed to 11 mm/s. The fraction of DRX in the matrix zone was higher than that in the welding zone, resulting in a smaller grain size in the matrix zone;
3. The expansion ratio first decreased from 1.25 at the ram speed of 3 mm/s to 1.15 at the ram speed of 7 mm/s due to the decrease in K* values, and then increased to 1.20 with the ram speed increasing to 11 mm/s, due to the uniform microstructure;
4. The welding qualities were determined not only by the thermodynamic parameters on the welding surface but also by the uniformity of the microstructure.

## Figures and Tables

**Table 1:** Crack propagation distances along the weld line.

Ram Speed (mm/s)	Distance (mm)				
	1	2	3	4	Average
7	0.75	0.93	0.68	0.87	0.81
9	0.73	0.79	0.67	0.63	0.71
11	0.65	0.60	0.70	0.68	0.66

## Data Availability

The primary data that support the results described here are available from the corresponding author upon reasonable request.
